# Patient’s Satisfaction with Hearing Aids: The Italian Version of the International Outcome Inventory for Hearing Aids (IOI-HA-It)

**DOI:** 10.3390/audiolres16010027

**Published:** 2026-02-14

**Authors:** Virginia Dallari, Enrico Apa, Silvia Palma, Chiara Gherpelli, Alberto Pisetta, Luca Sacchetto, Daniele Monzani

**Affiliations:** 1 Department of Otolaryngology Head and Neck Surgery, Santa Maria Delle Croci Hospital, AUSL Della Romagna, 48121 Ravenna, Italy; virginia.dallari@auslromagna.it; 2 Audiovestibology Unit, Department of Neuroscience, ASST Settelaghi, Varese Hospital, Viale Luigi Borri 57, 21100 Varese, Italy; enrico.apa@asst-settelaghi.it; 3 Audiology, Primary Care Department, AUSL of Modena, 41121 Modena, Italy; 4 Otorhinolaryngology and Audiology Unit, Department of Medical and Surgical Sciences for Children and Adults, Azienda Ospedaliero-Universitaria of Modena, 41125 Modena, Italy; 5 ENT, Department of Surgical Sciences, Dentistry, Gynaecology and Paediatrics, University of Verona, Borgo Roma Hospital, 37129 Verona, Italy; alberto.pisetta@univr.it (A.P.); luca.sacchetto@univr.it (L.S.); daniele.monzani@univr.it (D.M.)

**Keywords:** hearing aid, hearing loss, international outcome inventory for hearing aids

## Abstract

**Background**: Hearing aid (HA) outcome is a multidimensional construct that requires not only the analysis of auditory function improvement, but also a subjective evaluation of benefits from HAs. Indeed, subjective satisfaction of patients with HAs is not entirely predictable from audiometric outcomes such as real ear gain or functional gain. In light of this possible discrepancy the 1990 Consensus Statement for “Recommended Components of a Hearing Aid Selection Procedure for Adults” suggested that verification of hearing aids benefit also incorporate the subjective satisfaction with amplification. **Objectives**: The aim of this study was to test the validity and reliability of the Italian version of International Outcome Inventory for Hearing Aids (IOI-HA-It). **Methods**: Ninety-eight outpatients were randomly recruited to participate in this study. They all made regular use of HAs and were supplied with three different self-administered questionnaires. The International Outcome Inventory for Hearing Aids (IOI-HA), the Hearing Handicap Inventory for Adults (HHIA) or for elderly (HHIE) and the Italian translation of the MOS 36-Item Short Form Health Survey (SF-36). The epidemiological features and results were analyzed as descriptive statistics. Continuous variables were expressed as means with standard deviations (SDs). Reliability of the Italian version was assessed by the following two parameters: internal and test–retest consistencies. Internal consistency reliability was measured by Cronbach’s alpha coefficient. **Results and Conclusions**: This study evidenced that the IOI-HA-It is proved to offer adequate subjective outcome measures to better appreciate the integral evaluation of a patient’s rehabilitative experience. Furthermore, since it is a very brief questionnaire with low demand on time and cost involved in its compilation, it should be recommended in clinical practice.

## 1. Introduction

Hearing aid (HA) outcome is a multidimensional construct that requires not only the analysis of auditory function improvement, such as real ear gain, functional gain, speech intelligibility, and restoration of normal loudness growth, but also a subjective evaluation of the real benefit by the users [1,2]. Although these data are essential to the auditory rehabilitation process as they serve to verify the adequacy of the signal processing, their ability to appreciate the benefit from HAs is questionable [3]. For instance, audiometric tests may demonstrate an increase in speech intelligibility but hearing-impaired patients may not perceive a consensual disability and handicap reduction. Conversely, a little increase in the aided audibility could correspond to a great patient’s perceived benefit [4].

In light of this possible discrepancy the 1990 Consensus Statement for “Recommended Components of a Hearing Aid Selection Procedure for Adults” suggested that verification of hearing aids benefit also incorporate the subjective satisfaction with amplification [5]. Since then, the number of disease-specific questionnaires available for measuring patients’ satisfaction with hearing aid has increased. Despite the general agreement about the utility of such questionnaires, they are not yet used widely in clinical settings because they are often fairly time-consuming and costly. Besides this, a further reason for the HA outcome inventories are often impractical for research purposes is that most of them are not submitted to translation and validation in more than few languages. For this reason experimental results from different countries are hardly comparable. An important step in this direction was made by an international research team, who supported the concept of generating a brief universally applicable outcome measure [6]. The original English version of the International Outcome Inventory for Hearing Aids (IOI-HA) was therefore proposed and subsequently analyzed for its psychometric properties by Cox and Alexander [7]; thereafter, it was translated in more than 20 languages [8]. To the best of our knowledge, an exhaustive validation process of the Italian version has not yet been carried out as in a previous study, but it was just evaluated for reliability [9]. Therefore, the aim of this study was to test the validity and reliability of the Italian version of this international tool.

## 2. Materials and Methods

This prospective study was conducted at the Audiology Unit of the Otorhinolaryngology Unit of the University Hospital of Modena, Italy, from 2012 to 2020. It was approved by the Ethics in Research Committee of this Institution (260/09) on 15 December 2009. The experimental protocol followed the recommendations of the Declaration of Helsinki for Human Experimentation. Informed consent was obtained from each participant before examination and completion of questionnaires. A minimum sample size of 100 participants, or seven times the number of items in the questionnaire, has been indicated as an adequate sample size for questionnaire validation studies [10]. Based on standards for good methodological quality, this sample size has also been deemed adequate for the psychometric evaluation and validation of a questionnaire recommended in the Consensus-Based Standards for the Selection of Health Status Measurement Instruments (COSMINs) checklist [10]. HA adult users attending the Audiology Unit for periodic follow-up visits were selected using a random sampling method.

### 2.1. Selection Criteria

Only adult subjects have been enrolled.

Inclusion criteria were: regular use of hearing aids for at least six months before recruitment and a clear will to participate in the study. No segregation of subjects was made by type of hearing loss (sensorineural, conductive and mixed), type of hearing aids and any fitting variables.

Exclusion criteria were: not speaking or understanding the Italian language and the presence of major neurological or psychiatric disorders that could prevent the patients from filling in the questionnaires themselves.

A demographic information checklist including the variables of gender, age, employment status, education level, and type of hearing aid was used to collect the data.

### 2.2. Audiological Evaluation

In one session, all patients underwent free-field pure-tone and speech audiometry both aided and unaided, in order to determine the functional gain of their current hearing aids. Mean pure tone average thresholds (PTA) were calculated over 500, 1000, 2000 and 4000 Hz in both conditions. The degree of hearing loss was indicated according the ASHA’s classification as follows: normal hearing: PTA from −15 to 15 dB nHL; slight hearing loss: PTA from 16 to 25 dB nHL; mild hearing loss: PTA from 26 to 40 dB nHL; moderate hearing loss: PTA from 41 to 55 dB nHL; moderately severe hearing loss: PTA from 56 to 70 dB nHL; severe hearing loss: PTA from 71 to 90 dB nHL; and profound hearing loss: PTA from 91 dB nHL [11].

For the identification of the functional gain based on word recognition, the Italian version of the speech recognition threshold was used [12], determining the lowest intensity level at which the patient can correctly identify 50% of common two-syllable words. The patients were seated in a double wall, large booth and lists of words (5 lists of 20 common two-syllable recorded words) were presented to the subject being tested from one loudspeaker located at 0° in front of them at a distance of 1.5 m, at a fixed level of 70 dB. The competing noise was a ten-speaker multi-talker speech babble delivered by two other loudspeakers, located at 90° and 270°. The spectrum of multi-talker babbles was speech shaped. A signal-to-babble ratio of 10 dB was adopted [13].

Before audiometric tests started, subjects were supplied with the following three different self-administered questionnaires.

### 2.3. Questionnaires

*The International Outcome Inventory for Hearing Aids (IOI-HAs)* is a patient-reported outcome measures (PROMs) that consists of seven items, concerning the outcome domains of daily use (Use), benefit (Ben), residual activity limitations (RALs), satisfaction (Sat), residual participation restrictions (RPRs), impact on others (Ioth), and quality of life (QoL). Each item has five possible choices, on a Likert-scale, ranging from 1 (least favorable) to 5 (most favorable). Therefore, the score ranges from 5 to 35. High score indicates a benefit from HAs. The Italian version of IOI-HA published by Cox and Colleagues [8] was adopted (Appendix A) and firstly administered to a restricted sample of 20 patients, all HAs users (10 males and 10 females, aged between 45 and 65 years) with different social and cultural features in a face-to-face interview format. They were randomly selected among outpatients of our Audiological center. This procedure is useful to test face validity [14]. Since questions and wording were clearly understood by each patient, no lexical modification was performed.

*The Hearing Handicap Inventory for Adults (HHIAs)* [15] and the Hearing Handicap Inventory for the Elderly (HHIE) [16]. Both questionnaires are widely used and composed of a 13-item emotional subscale and a 12-item socio-situational subscale that were designed to assess emotional reaction and social limitations perceived by hearing-impaired subjects. The HHIA and the HHIE should be used in patients aged below and over 65 years respectively. HHIA was derived from HHIE and two replacement questions from the HHIE focus on the occupational effects of hearing loss (E7-S11) since younger adults are more often occupied in work than the elderly who are more frequently retired. A *yes* response to an item was awarded 4 points, *sometimes* 2 points and a *no* 0 points. Therefore, scores range from 0 to 100 points, indicating an increasing level of perceived handicap in both questionnaires. In the framework of this investigation, only the HHIA-It [17] was used regardless of the patients’ age since all HAs users undertook ordinary work at the time of examination.

*The Italian translation of the MOS 36-Item Short Form Health Survey (SF-36)* was used [18] to further investigate the psycho-sociological domains of health-related quality of life. This scale comprises 36 items subdivided into the following eight health subscales: general health (GH), physical functioning (PF), role-physical (RP), bodily pain (BP), vitality (V), social functioning (SF), role-emotional (RE) and mental health (MH). Each dimension is separately scored using item weighting and additive scaling. Summed data were then transformed into a 0 to 100 points scale, higher score indicating better health. Only the social functioning (SF) and the role-emotional (RE) subscales were employed in this investigation, in analogy to the previous Italian validation study of the HHIA [14]. The first one measures the impact of physical and emotional problems on social activities (with family, friends, and groups of people), while the second rates the interference of psychological distress (such as feeling anxious and/or depressed) with work and other ordinary daily activities.

### 2.4. Statistical Analysis

The epidemiological features and results were analyzed as descriptive statistics. Continuous variables were expressed as means with standard deviations (SDs). Reliability of the Italian version was assessed by the following two parameters: internal and test–retest consistencies. Internal consistency reliability was measured by Cronbach’s alpha coefficient [19]. This procedure is helpful in deciding whether different questions in a questionnaire are measuring the same underlying concept; in this case, whether each item of the scale is a consistent indicator of hearing aids benefit. It is calculated using a one-way analysis of the variance model with items functioning as the repeated measure. Cronbach’s α ranges from 0 to 1, with higher values indicating greater internal consistency or reliability of the survey. A Cronbach’s alpha coefficient of 0.70 is the minimally acceptable level for internal consistency reliability [20]. It was additionally computed by Cronbach’s α if each of the items was deleted. This procedure determines through appropriate correlations the degree of inner consistency in case any single item was excluded from the scale. As a fact we used Cronbach’s α mainly to compare internal consistency of the IOI-HA-It with those of the original English version and other validated European ones. Anyway, latest statistical studies indicate that Cronbach’s α also depends on the number of items of the scale (the lower the number of items, the lower the α coefficient) so that another reliability coefficient, the McDonald’s ω, has been recently introduced and adopted in this study [21]. Unlike the α coefficient, the ω coefficient works with the load factorials that are the weighted sum of the variables standardized, a transformation that generates more stable calculations and reflects the true level of reliability. In addition, it does not depend on the number of items.

Spearman’s Coefficient ρ was calculated in order to assess test–retest reproducibility and Kendall’s τ to correlate IOI-HA-It score with the score of the other validated questionnaires in order to test convergent validity. The level of statistical significance was considered reached if *p*-value was <0.05 in all procedures. The Statistical Package for the Social Sciences (IBM SPSS^®^ version 25.0 for Microsoft Windows^®^) was applied for statistical analysis and graphical representation.

## 3. Results

A total of 105 HA users were selected using a random sampling method. Seven patients invited to take part in the study returned the questionnaires not correctly completed and were therefore excluded from this study. Therefore, the sample consisted of 98 outpatients (age 18 years and over) attending the Audiology Unit for periodic follow-up visits. A subgroup of 40 patient was randomly selected for test–retest analysis conducted by phone interview as follows: 5 patients could not be reached, so the sample was definitely constituted of 35 subjects. Then, 61 (62.2%) were males and 37 (37.8%) females (Table 1). The mean age was 58.7 years (SD: 12.7; range: 18–72), whereas the mean value of PTA unaided was 55.9 dB nHL (SD: 8.1; range: 36.2–67.5 dB nHL) and with hearing aids was 35.2 dB nHL (SD: 6.8; range: 19–53 dB nHL). Most participants (89%) were fitted bilaterally. All HAs were at the minimum feature settings with digital feedback reduction, digital noise reduction and automatically activated fixed directional systems. Almost all devices, except in six cases, incorporated some type of advanced processing such as automated learning algorithms, systems to analyze sound environments and blend features in real-time, voice priority processing. No cases used HA with deep neural networks. As regards connectivity, all hearing aids but eight enabled patients with direct streaming for calls while Bluetooth technology streamers were exceedingly rare (three cases).

Eighty-nine patients were fitted with BTE (behind the ear) instruments while nine wore ITE (in-the-ear) devices.

In the unaided condition all participants reached the speech recognition threshold (on average at 65.7 dB nHL; SD: 12.6; range: 40–80 dB nHL).

With hearing aids all subjects reached the speech recognition threshold (on average at 45.3 dB nHL; SD: 10.4; range: 25–60 dB nHL). The functional gain, intended as the difference between aided and unaided condition, was on average 18.6 dB nHL (SD = 11.5, range 15–45). This functional gain refers to the difference between SRT in the two conditions.

Considering PTA in the better ear in unaided condition, two patients (2.0%) had mild hearing loss, 23 (23.5%) had a moderate hearing loss and 73 (74.5%) had a moderately severe hearing loss; the results after HA rehabilitation are also reported.

### 3.1. IOI-HA’s General Properties

The correlations among the items of the instrument are shown in Table 2. Significant correlations were observed between Sat, Ben, RPR and QoL, between Ben and Use, Sat, and in addition between RAL and Sat. Significant correlations were observed between each item and the total score.

### 3.2. Internal Consistency and Reproducibility

Main psychometrics properties of the IOI-HA are reported in Table 3. The Cronbach’s α coefficient was 0.736, whereas the McDonald’s ω coefficient was 0.759. In the procedure ‘Cronbach’s α if item is deleted’, the overall α coefficient was not improved by the removal of any item. The test–retest reliability, evaluated by Spearman’s correlation coefficient ρ, was found to be correlated at a significant level for the total score and each item (Table 3). The floor effect was 2.0% for RAL and 0.0% for all other items and the total score. In contrast, although the ceiling effect was above 15% for items 1 and 4, for the total score it was 8.2%.

### 3.3. Construct Validity

The assessment of the Kendall’s τ coefficient reported significant correlations between the subscale RE of the SF-36 and Ben, RPR, Ioth, IOI-HA total score and QoL; between SF and total score of IOI-HA; and between this subscale of the SF-36, Ben and Qol. Significant correlations were also reported between SF and Sat and Ioth. Finally, significant correlation was reported between MH and QoL and between this subscale of SF-36 and Ben, RAL, Sat, RPR and total score.

### 3.4. Criterion-Related Validity

Negative significant correlations were found between the HHIA and most of the IOI-HA items and its total scores (τ = −0.502; *p* < 0.01). In detail, significant correlations were reported between HHIA and Ben (τ = −0.424; *p* < 0.01), RAL (τ = −0.423; *p* < 0.01), Sat (τ = −0.440; *p* < 0.01), and QoL (τ = −0.458; *p* < 0.01). A significant correlation was reported between HHIA and RPR (τ = −0.458; *p* < 0.01). Finally, the main psychometrics properties of the IOI-HA in the present study, compared to the ones of other European validation studies, are reported in Table 4.

## 4. Discussion

The primary endpoint of this study was to test the validity and reliability of the Italian version of the IOI-HA, an international tool, whose diffusion and use worldwide is increasing more and more.

The Cronbach’s α coefficient was 0.736, very similar to both the original English and Dutch version but a little lower than those computed in a recent Italian investigation about hearing aids stigma [9] that resulted 0.84 and of the French validation study which reported a Cronbach’s α of 0.86 [26]. More than one explanation could be supposed to explain these differences such as age of patients enrolled, different social and emotional engagement, different technologies of hearing aids and limited connectivity. Moreover, as Kramer showed in her investigation about IOI-HA in the Netherlands, Cronbach’s α could significantly vary depending on the type of hearing loss being high values described for conductive and the worst for neurosensorial type [22]. It could also depend on the fact that patients could be first time users or habitual. Moreover, in this latest study only Cronbach’s α a was calculated in the absence of test–retest reliability and convergent validity to other disease-specific questionnaires measuring hearing disability [9].

Furthermore, the statistical procedure ‘Cronbach’s α if item is deleted’ was used to check whether removing items from the scale improves the overall α coefficient. As a fact, the overall α coefficient was not improved by the removal of any item, thus indicating that all items forming the scale measure a latent variable in common, namely hearing aid outcome. Finally, the ceiling and floor effects were calculated by percentage frequency of highest or lowest possible score achieved by respondents respectively. If present, a percentage more than 15%, such in the case for items 1 and 4, indicates that reliability is reduced, due to the difficulty in distinguishing patients with the lowest or highest possible score from each other. This clustering at the top for item 1 score is reasonably due to the fact that HA use increases directly proportionate to the degree of hearing loss. The high percentage of moderately severe hearing loss patients in this study contributes to this ceiling effect [23]. Anyway, when assessing ceiling effects in questionnaires, both individual items and the total score are crucial: item-level analysis reveals which specific questions are too easy (leading to clustering at the top); total scores not clustering at the top mean the entire instrument is able to capture nuances in high-achievement/satisfaction groups.

In this study this percentage was 8.2 for the total score and therefore significant bunching of scores at the upper and lower levels reported by this instrument was excluded and no attenuation effect of the scale identified [27].

McDonald’s ω coefficient in addition to Cronbach’s α coefficient was also computed as it estimates reliability from a factor analysis framework, not being influenced by the number of items. In this study, α was slightly smaller than ω coefficient, in accordance with other studies that computed both coefficients to estimate reliability [28]. Reproducibility is crucial for measuring the stability of scores over time. It indicates the likelihood of obtaining the same or similar scores if the questionnaire is administered again to the same sample. The test–retest method was adopted, comparing the scores in a repeated administration after an interval of six weeks to 35 selected subjects who had no change in their hearing ability with HAs. This time interval was intended to minimize the patients’ recall of the first compilation of the questionnaire that might increase correlation between the two administrations [29]. The IOI-HA was administered again during follow-up visits and a significant Spearman’s coefficient ρ was reported for all items and the total score of the scale, thus indicating a good rest–retest reproducibility. These results suggest that IOI-HA-It should be regarded as a reliable tool not only to appreciate the multidimensional aspects of aural rehabilitation in the first assessment of hearing aids benefits but also to appreciate further patients’ improvement satisfaction with their hearing aids as time goes by or, conversely, to adopt more convincing amplification parameters if benefit perception decreases.

The construct validity refers to the ability of an instrument to measure a theoretically derived hypothesis. Thus, the Kendall’s τ coefficient was used to disclose the presumed correlation between the RAL and RPR domains of IOI-HA and the SF and RE subscales of the SF-36. Similarly, correlation between the QoL dimension of IOI-HA and the MH subscale of the SF-36 was examined. As a fact, all these variables resulted positively correlated so that IOI-HA-It came to represent not only a valid disease-specific measure but also a useful instrument to assess the multiple psychological and social aspects of aural rehabilitation. This attribute of the IOI-HA-It was further confirmed by Kendall’s τ coefficient that clearly showed significant and negative correlations between Ben, Sat, RAL and RPR of the IOI-HA and the HHIA. It was therefore documented that a decreasing level of perceived hearing disability is related to self-perceived success of the rehabilitation program with hearing aids, as expected. On the contrary, no significant correlation between IOI-HA-It total score and the functional gain was found. This result seems to resemble those of a previous study that stated how technical audiological data alone cannot predict the subject’s perception of benefit and satisfaction from HA use.

This study has some limits. The first one is that most patients enrolled (74.5%) were affected by moderately severe hearing loss and a lower percentage by a mild degree. It should be noticed that when this study starts according to MarkeTrak VIII, only 1/10 cases with mild hearing loss get HAs compared with 4/10 of their peers with moderate-to-severe impairments [30]. These data were also confirmed in 2018 by Carole Johnson [31]. Furthermore, in Italy the Health Care System does not provide hearing impaired subjects with financial support to purchase hearing aids before the PTA in the better ear is greater than 55 dB HL. Taken together these data could account for the lower percentage of patients with mild to moderate hearing loss who do not purchase and use hearing aids so that IOI-HA-it can be largely applied to the majority of HA users (with moderately severe hearing loss or worse). Further investigation into the survey specifically targeted to slight to mild hearing loss and related HA satisfaction and benefit are needed.

Another limit could be identified in the apparent small sample size of subjects enrolled to test–retest reliability. As a fact there is no universally accepted standard to indicate the adequacy of sample size to test–retest reliability of a questionnaire. For example, Bonnet & Wright asserted that samples must be as small as thirty to establish reliability so long as the scale items have strong inter-correlation [32] and Rea & Parker posited that smaller samples may suffice for test–retest reliability [33]. Another theory stated that test–retest reliability should be based on a sample size of 400 patients at least [34]. As a rule of thumb, also in the French validation process of IOI-HA translation, the authors adopted a sample of 35 patients for test–retest [26].

Finally, the sample size was 98 subjects instead of the 100 planned. We considered that it represents a negligible risk, as the drop-off in precision (margin of error) is minimal. The *real* problem could come if the sample size was *too small* for the specific analysis, severely impacting the reliability. Ninety-eight is generally acceptable for basic measures like Cronbach’s alpha especially under specific conditions like strong item correlations such as in this case.

## 5. Conclusions

HAs are reported to be effective at improving listening ability and hearing-specific health-related quality of life, which is an increase in social activity and a reduction in participation restriction. The increasing interest in assessing the impact of aural rehabilitation on these domains is a reason to select appropriate outcome measurement tools. The emerging importance of adequate subjective outcome measures to better appreciate the integral evaluation of a patient’s rehabilitative experience also corresponds to the necessity to provide a cost–benefit analysis to justify allocation of health-care financial resources. The IOI-HA, Italian version, is proven to possess these characteristics. Furthermore, since it is a very brief questionnaire with low demand on time and cost involved in its compilation, it should be recommended in clinical practice. Finally, a further reason for the use of IOI-HA-It is its good reproducibility so it can be used to test the advantages of new technologies and/or therapies.

## Figures and Tables

**Table 1:** Participant characteristics. SD = standard deviation; min–max, lowest and highest values; PTA, pure tone average threshold (500 Hz, 1000 Hz, 2000 Hz, 4000 Hz) in the better ear; SRT = Speech Recognition Threshold (the minimum hearing level at which an individual can correctly repeat 50% of bisyllabic words at speech audiometry).

Variable	Total (n = 98)			Male (n = 61)			Female (n = 37)		
	Mean	SD	Min–Max	Mean	SD	Min–Max	Mean	SD	Min–Max
Age (years)	58.7	12.7	18–72	57.5	13.5	18–70	60.6	11.3	33–72
PTA (dB nHL)	55.7	8.9	36.5–70	55.7	8.9	36.5–70	54.7	7.6	35–70
PTA (dB nHL) with hearing aids	35.2	6.8	19–53	36.1	6.6	26–53	33.7	6.9	19–46
SRT (dB nHL)	65.7	12.6	40–93	66.8	12.6	40–88	64.0	12.4	48–93
SRT (dB nHL) with hearing aids	49.1	10.4	25–70	51.5	9.5	35–70	48.4	11.6	25–70
Educational status	30 (30.6%) Primary school 30 (30.6%) Secondary school 25 (25.6%) High school 13 (13.2%) Academic			14 (23.0%) Primary school 20 (32.8%) Secondary school 20 (32.8%) High school 7 (7.4%) Academic			16 (43.2%) Primary school 10 (27.0%) Secondary school 5 (13.6%) High school 6 (6.2%) Academic		

**Table 2:** Inter-item correlation of the IOI-HA. Use = daily use; Ben = benefit; RAL = residual activity limitations; Sat = satisfaction; RPR = residual participation restrictions; Ioth = impact on others, QoL = quality of life. Cronbach’s α is sensitive to the correlations between items on a scale, the higher the inter-items correlation the higher the α. * *p* < 0.05; ** *p* < 0.01.

Item n.		1	2	3	4	5	6	7
		Use	Ben	RAL	Sat	RPR	Ioth	QoL
1	Use		0.471 **	0.084	0.303 **	0.107	0.007	0.391 **
2	Ben			0.287 **	0.551 **	0.248 *	0.267 *	0.508 **
3	RAL				0.400 **	0.131	−0.013	0.295 **
4	Sat					0.250 *	0.169	0.491 **
5	RPR						0.250 *	0.482 **
6	Ioth							0.312 **
7	QoL							
	Total	0.405 **	0.609 **	0.415 **	0.680 **	0.407 **	0.342 **	0.648 **

**Table 3:** Main psychometrics properties of the IOI-HA. Cronbach’s α coefficient and McDonald’s ω are reported. Cronbach’s α “if item is deleted” is reported and confirms that all items are intended to explore the same domains. Floor and ceiling effects show that the first is near 0 and the second varies from 2% to 80.6%. Spearman’s ρ coefficient is reported to test reproducibility. * *p* < 0.05; ** *p* < 0.01. Use = daily use; Ben = benefit; RAL = residual activity limitations; Sat = satisfaction; RPR = residual participation restrictions; Ioth = impact on others, QoL = quality of life.

Item n.		Cronbach’s α Coefficient	McDonald’s ω Coefficient	Cronbach’s α “If Item Is Deleted”	Floor Effect	Ceiling Effect	Reproducibility (n = 35)
							Spearman’s ρ Coefficient
1	Use	0.736	0.759	0.722	0.0%	80.6%	0.284 *
2	Ben			0.692	0.0%	3.1%	0.401 **
3	RAL			0.715	2.0%	2.0%	0.266 *
4	Sat			0.692	0.0%	45.9%	0.325 *
5	RPR			0.724	0.0%	1.0%	0.234 *
6	Ioth			0.729	0.0%	3.1%	0.385 **
7	QoL			0.677	0.0%	5.1%	0.588 **
	Tot	-	-	-	0.0%	8.2%	0.702 **

**Table 4:** Mean and standard deviation IOI-HA single item mean in the present and other validation studies. IOI-HA, the International Outcome Inventory for Hearing Aids; Use, daily use; Ben, benefit; RAL, residual activity limitations; Sat, satisfaction; RPR, residual participation restrictions; Ioth, impact on others QoL, quality of life; Tot, total score; -, not explored.

Item n.		Present Study	Cox [8]	Kramer [22]	Brännström [23]	Paiva [24]	Heuermann [25]
		(n = 98)	(n = 172)	(n = 505)	(n = 380)	(n = 80)	(n = 80)
1	Use	4.78 (±0.49)	3.73 (±1.17)	4.34 (±0.99)	3.9 (±1.1)	4.54 (±0.84)	4.6 (±0.7)
2	Ben	3.59 (±0.59)	3.39 (±0.98)	3.19 (±1.39)	4.0 (±1.1)	3.88 (±1.02)	3.7 (±1.0)
3	RAL	3.15 (±0.74)	3.40 (±0.95)	3.51 (±1.15)	3.5 (±1.2)	3.19 (±1.04)	3.5 (±1.0)
4	Sat	4.42 (±0.57)	3.20 (±1.21)	3.61 (±1.21)	4.3 (±1.0)	4.08 (±1.04)	3.8 (±1.3)
5	RPR	3.60 (±0.53)	3.57 (±1.13)	3.71 (±1.14)	4.1 (±1.1)	3.91 (±1.02)	3.9 (±0.9)
6	Ioth	3.74 (±0.54)	3.79 (±1.13)	3.84 (±1.06)	3.9 (±1.1)	3.98 (±1.13)	4.5 (±0.9)
7	QoL	3.65 (±0.64)	3.19 (±0.93)	3.25 (±1.26)	3.8 (±1.1)	3.75 (±0.97)	3.6 (±1.1)
Cronbach’s α coefficient		0.736	0.78	0.77–0.78	0.8–0.81	0.838	0.84
Reproducibility		0.81	-	0.62–0.73	-	-	-

## Data Availability

All data generated or analyzed during this study are included in this article. Further enquiries can be directed to the corresponding author.

## Appendix A. The Italian Version of the International Outcome Inventory for Hearing Aids (Retrieved by D. Cuda)

1. Pensi a quanto ha usato la sua attuale protesi acustica nelle ultime due settimane. In media, al giorno, quante ore ha usato la protesi?				
□ Nessuna	□ Meno di un’ora al giorno	□ 1–4 ore al giorno	□ 4–8 ore al giorno	□ Più di 8 ore al giorno
2. Pensi ad una situazione nella quale avrebbe volute sentire meglio, prima di avere la sua attuale protesi acustica. Nelle ultime due settimane, quanto l’ha aiutata la sua protesi in quella situazione?				
□ Per niente	□ Un po’	□ Moderatamente	□ Abbastanza	□ Molto
3. Pensi ancora alla situazione nella quale avrebbe volute sentire meglio. Quando utilizza la sua attuale protesi acustica, quanta difficoltà prova ancora in quella situazione?				
□ Molto	□ Abbastanza	□ Moderatamente	□ Un po’	□ Per niente
4. Considerando tutto, pensa che la sua attuale protesi acustica riesca a risolvere il suo problema d’udito?				
□ Per niente	□ Un po’	□ Moderatamente	□ Abbastanza	□ Molto
5. Nelle ultime due settimane, con la sua attuale protesi acustica, ha avuto difficoltà a fare ciò che voleva?				
□ Molto	□ Abbastanza	□ Moderatamente	□ Un po’	□ Per niente
6. Nelle ultime due settimane, con la sua attuale protesi acustica, in che misura pensa che le altre persone siano state infastidite dalla sua difficoltà di udito?				
□ Molto	□ Abbastanza	□ Moderatamente	□ Un po’	□ Per niente
7. Considerando tutto, quanto la sua attuale protesi acustica ha cambiato in positive la sua vita?				
□ In peggio	□ Per niente	□ Un po’	□ Abbastanza	□ Molto

## References

[1] Humes L. (1999). Dimensions of hearing aid outcome. J. Am. Acad. Audiol..

[2] Ricketts T., Van Vliet D. (1996). Updating our fitting strategies given new technology. Am. J. Audiol..

[3] Saunders G.H., Chisolm T.H., Abrams H.B. (2005). Measuring hearing aid outcomes--not as easy as it seems. J. Rehabil. Res. Dev..

[4] Weinstein B.E. (1997). Outcome measures in the hearing aid fitting/selection process. Trends Amplif..

[5] Hawkins D.B., Beck L.B., Bratt G.W., Fabry D.A., Mueller H.G., Stelmachowicz P.G. (1991). Vanderbilt/VA Hearing Aid conference 1990 consensus statement. Recommended components of a hearing aid selection procedure for adults. Asha.

[6] Cox R., Hyde M., Gatehouse S., Noble W., Dillon H., Bentler R., Stephens D., Arlinger S., Beck L., Wilkerson D. (2000). Optimal outcome measures, research priorities and international cooperation. Ear Hear..

[7] Cox R.M., Alexander G.C. (2002). The International Outcome Inventory for Hearing Aids (IOI-HA): Psychometric properties of the English version. Int. J. Audiol..

[8] Cox R.M., Stephen D., Kramer S.E. (2002). Translations of the International Outcome Inventory for Hearing Aids (IOI-HA). Int. J. Audiol..

[9] Apa E., Ferrari S., Monzani D., Ciorba A., Sacchetto L., Dallari V., Nocini R., Palma S. (2025). Body Image Concerns and Psychological Distress in Adults with Hearing Aids: A Case-Control Study. Audiol. Res..

[10] Terwee C.B., Mokkink L.B., Knol D.L., Ostelo R.W.J.G., Bouter L.M., de Vet H.C.W. (2012). Rating the Methodological Quality in Systematic Reviews of Studies on Measurement Properties: A Scoring System for the COSMIN Checklist. Qual. Life Res..

[11] Clark J.G. (1981). Uses and abuses of hearing loss classification. Asha.

[12] Turrini M., Cutugno F., Maturi P., Prosser S., Leoni F.A., Arslan E. (1993). Bisyllabic words for speech audiometry: A new italian material. Acta Otorhinolaryngol. Ital..

[13] Monzani D., Nocini R., Presutti M.T., Gherpelli C., Di Berardino F., Ferrari S., Galeazzi G.M., Federici G., Genovese E., Palma S. (2022). The Effect of the Use of Hearing Aids in Elders: Perspectives. Audiol. Res..

[14] Holden R.B., Weiner I.B., Craighead W.E. (2010). Face Validity.

[15] Newman C.W., Weinstein B.E., Jacobson G.P., Hug G.A. (1990). The Hearing Handicap Inventory for Adults: Psychometric Adequacy and Audiometric Correlates. Ear Hear..

[16] Ventry I.M., Weinstein B.E. (1982). The hearing handicap inventory for the elderly: A new tool. Ear Hear..

[17] Monzani D., Genovese E., Palma S., Rovatti V., Borgonzoni M., Martini A. (2007). Measuring the psychological consequences of hearing loss in a working adult population: Focus on validity and reliability of the Italian translation of the Hearing Handicap Inventory. Acta Otorhinolaryngol. Ital..

[18] Apolone G., Mosconi P. (1998). The Italian SF-36 Health Survey: Translation, validation and norming. J. Clin. Epidemiol..

[19] Cronbach L. (1951). Coefficient alpha and the internal structure of the test. Psychometika.

[20] Nunnally J.C. (1978). Psychometric Theory.

[21] Cho E. (2016). Making reliability reliable: A systematic approach to reliability coefficients. Organ. Res. Methods.

[22] Kramer S.E., Goverts S.T., Dreschler W.A., Boymans M., Festen J.M. (2002). International Outcome Inventory for Hearing Aids (IOI-HA): Results from The Netherlands. Int. J. Audiol..

[23] Brännström K.J., Wennerström I. (2010). Hearing aid fitting outcome: Clinical application and psychometric properties of a Swedish translation of the international outcome inventory for hearing aids (IOI-HA). J. Am. Acad. Audiol..

[24] Paiva S.M., Simões J.F., Paiva A.M., Sousa F.J., Bébéar J.P. (2017). Translation of the international outcome inventory for hearing aids into Portuguese from Portugal. BMJ Open.

[25] Heuermann H., Kinkel M., Tchorz J. (2005). Comparison of psychometric properties of the International Outcome Inventory for Hearing Aids (IOI-HA) in various studies. Int. J. Audiol..

[26] Tuset M.-P., Daval M., Levy D., Ayache D., Gargula S. (2025). French Adaptation and Validation of the International Outcome Inventory on Hearing Aids (IOI-HA) Questionnaire. Audiol. Res..

[27] Vogt W.P., Burke Johnson R., Vogt W.P. (2005). Scale Attenuation Effect. Dictionary of Statistics & Methodology.

[28] Marcusson-Clavertz D., Kjell O.N. (2019). Psychometric properties of the spontaneous and deliberate mind wandering scales. Eur. J. Psychol. Assess..

[29] Demorest M., Walden B. (1984). Psychometric principles in the selection, interpretation, and evaluation of communication self-assessment inventories. J. Speech Hear. Disord..

[30] Kochkin S. (2012). MarkeTrak VIII: The key influencing factors in hearing aid purchase intent. Hear Rev..

[31] Johnson C.E. (2018). The Early Intervention of Hearing Loss in Adults. Semin. Hear..

[32] Bonett D.G., Wright T.A. (2015). Cronbach’s alpha reliability: Interval estimation, hypothesis testing, and sample size planning. J. Organ. Behav..

[33] Rea M.L., Parker R.A. (1992). Designing and Conducting Survey Research: A Comprehensive Guide.

[34] Charter R.A. (1999). Sample Size Requirements for Precise Estimates of Reliability, Generalizability, and Validity Coefficients. J. Clin. Exp. Neuropsychol..

